# Impact of the Non-Contributory Social Pension Program *70 y más* on Older Adults’ Mental Well-Being

**DOI:** 10.1371/journal.pone.0113085

**Published:** 2014-11-19

**Authors:** Aarón Salinas-Rodríguez, Ma. Del Pilar Torres-Pereda, Betty Manrique-Espinoza, Karla Moreno-Tamayo, Martha María Téllez-Rojo Solís

**Affiliations:** 1 Center for Evaluation Research and Surveys, National Institute of Public Health, Cuernavaca, Mexico; 2 Center for Health Systems Research, National Institute of Public Health, Cuernavaca, Mexico; Federal University of Rio de Janeiro, Brazil

## Abstract

**Background:**

In 2007, a non-contributory pension program was launched in rural areas of Mexico. The program consisted in a non-conditional cash transfer of US$40 monthly to all older adults (OA) aged 70 and over. We evaluate the effect of the program on mental well-being of its beneficiaries.

**Methods and Findings:**

Quantitative and qualitative methods were used. For the quantitative component, we used the selection criteria established by the program (age and locality size) to form the Intervention (OA aged 70–74 residing in rural localities, <2500 inhabitants) and Control groups (OA aged 70–74, in localities with 2501–2700 inhabitants). Baseline data collection was conducted in 2007 where 5,465 OA were interviewed. The follow-up survey was conducted in 2008, and it was possible to interview 5,270 OA, with a response rate of 96%. A difference-in-difference linear probability model with individual fixed effect was used to estimate the impact of the program on mental well-being indicators. In 2009 a qualitative component was designed to explore possible causal pathways of such effect.

**Results:**

After a year of exposure, the program had a significant effect on reduction of depressive symptoms (β = −0.06, CI_95%_ −0.12; −0.01) and an increase in empowerment indicators: OA participated in important household decisions (β = 0.09, CI_95%_ 0.03;0.15); and OA participated in household decisions pertaining to expenses (β = 0.11, CI_95%_ 0.05;0.18). Qualitative analysis found a strong trend showing a reduction of sadness, and feeling of increasing empowerment.

**Conclusions:**

These results suggest that a non-conditional transfer in older ages have an impact beyond the economic sphere, impacting even the mental well-being. This effect could be explained because the pension produces feelings of safety and welfare. It is recommendable that governments should invest efforts towards universalizing the non-contributory pension programs in order to ensure a basic income for the elderly.

## Introduction

One of the most important demographic challenges expected for low and middle-income countries in the 21st century will be the increase of the number of older adults (OA) and the pressure this will have on social security systems, available medical assistance, and service demand for elderly care. Moreover, estimates of health problems and disability suggest OA in these countries are aging with more functional limitations and worse health conditions than OA in developed countries [1].

Aging process in low and middle-income countries is characterized by the presence of poverty and inequality. In these countries, poverty among OA (60 years and over) is higher than that for the entire population [2]. Because of this, old age could be a stage of life characterized by the reduction of formal work activities that in turns leads to a decrease in income and, consequentially, economic insecurity. Income insecurity in old age may have a negative effect on the welfare of the elderly and can often cause the impoverishment of the household.

Poverty in old age is also an important problem in low and middle-income countries. Studies in 15 low-income countries in sub-Saharan Africa report households with an elderly individual had higher levels of poverty that general population [3]. In particular, Latin America rates of poverty among the elderly range from 9.8% in Chile to 69.9% in Honduras [4]. More recent data from a study by CEPAL in 2008 reveals that in nine of 15 countries surveyed, more than 30% of the OA are poor. In Mexico, the incidence of poverty among the elderly population is about 30% [5].

In terms of income distribution, Latin America is considered the world's most imbalanced region [2]. Inequality is reflected in a significant number of socioeconomic dimensions, including access to social protection systems, in which Latin America's largest concern is its low coverage [2]. Economic insecurity affects OA living in poverty, but especially to those who formerly worked in the informal sector or were unpaid workers. Globally, it is estimated that four out of five elderly individuals have no pension or retirement, which forces the elderly to continue working and/or to depend on informal social support networks to subsist [6].

The majority of uninsured and retired individuals lives and works in low and middle-income countries. In Latin America, over 30% of the OA do not report retirement, pension, or employment income [5]. Many of them also do not participate in pension plans since they are unpaid caregivers, unemployed, or are employees in agriculture or in the informal work sector [7]. According to data from the survey CEPAL 2010 in Mexico, only about a quarter of OA receive benefits from the social security system through a social or retirement pension. In the richest quintile of the population, the coverage reaches 50%; however, in the poorest quintile, this number does not even reach 3% [8].

### Non-contributory social pensions

The rapid growth of the OA population in low and middle-income countries and the low coverage of social security reinforce the need to adapt social protection systems at a faster rate than the developed countries did [2]. One example is the implementation of social pensions for the elderly in order to reduce poverty. Currently, there is documented experience in countries like Nepal, Lesotho, Brazil, and South Africa, which are among the 80 countries that have established social pensions in the world. Of these, 47 are low- and middle-income countries, such as Mexico [6].

The most distinctive characteristics of noncontributory social pensions are that they are designed to be accessible as a right to all those who meet the requirements of the program and that the grant conditions are unrelated to work experience or the history of the market [9]. Noncontributory pension programs provide cash benefits relatively uniformly in a targeted or categorical manner to reduce the risks of old age and disability. In some countries, these programs also focus on reducing the risks of disease, as well as on providing a way to access other benefits of the social protection system (e.g. family allocations). In general, these programs provide modest and relatively uniform benefits [10], and it is considered that these programs are useful tools for women and individuals from the informal sector of the economy, who have benefited less by the contributory retirement system [7].

The results from the evaluation studies suggest that noncontributory social pension programs have had a major impact in reducing poverty in old age, as well as the incidence of extreme poverty, and a positive effect on reducing indigence [10]. Reports from Brazil and South Africa, two countries with the highest noncontributory pension programs, report that these programs not only are efficient vehicles to reduce poverty, but also have an influence on the magnitude rather than simply the incidence of poverty. Households with a noncontributory pension beneficiary have greater financial stability and are less likely to experience a decline in their living standards. Also, receiving the pension is associated with investments in human, physical, and social capital, in addition to being able to combat gender inequality [4], [11]–[16]. Also there has been some evidence about the effect of pensions on subjective well-being [17]–[19]. However, evaluations mainly have focused on studying the effect of these programs on income and poverty, and still little is known about their effects on other facets of OA life, such as physical health and mental health.

Given the currently gap in the evaluation literature regarding the impact of economic transfer programs on the OA in other areas more than just economic effects, our objective was to estimate the impact of the non-contributory pension program *70 y más* on mental well-being of its beneficiaries.

### The Program 70 y más

Implemented nationally throughout Mexico, Program *70 y más* was aimed at improving the living conditions among adults aged 70 years and older by boosting their social protection through policy mechanisms. Centered on two components, *70 y más* pursues a twofold objective: (1) to raise the income of the elderly and (2) to improve the social protection of the elderly. At the start of the program in 2007, the program had enrolled a total of one million beneficiaries and had a total annual budget of 6,250 million Mexican pesos (approximately US$595 million). In 2009 the number of beneficiaries had grown to 1.8 million, and the total budget had more than doubled to 13,000 million Mexican pesos (US$1,400 million) [8]. For the most recent data from 2014, the number of beneficiaries will be 3.9 million, with a total budget of 45,200 million Mexican pesos (US$3,476 million) [20].

Under the first program objective, OA receive a direct unconditional cash transfer of 500 Mexican pesos (approximately US$40) every month, which can be collected every two months. At the start of the program in 2007, the established eligibility criteria included being 70 years and over and residing in a locality with 2,500 or less inhabitants (rural localities). It should be noted that our evaluation is based on the two eligibility criteria and does not account for the fact that the program expanded the eligibility criteria to those residing in a locality up to 30,000 inhabitants after 2011 [21]. Today, and with a new government, the program has expanded even until some poor urban localities (>2500 inhabitants) and reduced the age range (≥65 years) of its beneficiaries.

The second program objective related to health-oriented social participation and social protection actions was late to start, and during the period of this evaluation, it only had enrolled a very small percentage of beneficiaries. For that reason, this work will focus on evaluating the impact of the program's cash transfer feature only.

## Methods

### Ethics Statement

The Research and Ethics Committees of Mexico's National Institute of Public Health approved the original study. Participants received a detailed explanation of the procedures and signed an informed consent declaration before data collection occurred.

The estimated impact of *70 y más* was derived from both quantitative and qualitative analyses of data. Both components (quantitative and qualitative) of the study are briefly presented in the next paragraphs.

### Quantitative component

In 2007, at the start of the program, *70 y más* established two eligibility criteria: (1) beneficiaries had to be 70 years old and over and (2) had to reside in localities of 2,500 or less inhabitants. We used these eligibility criteria to design our impact evaluation study and to identify the group exposed to the program (70–74 years of age, in localities with 2,500 or less inhabitants) and to establish three control groups: Group 1 (aged 65–69, in localities with 2,500 or less inhabitants); Group 2 (aged 70–74, in localities with 2,501–2,700 inhabitants), and Group 3 (aged 65–69, in localities with 2,501–2,700 inhabitants). Details about intervention and control group selection can be found in Appendix S1 in File S1.

#### Sample size, power calculations, and surveys

Sample size was determined according to 35 indicators related to household characteristics, household expenditures, health conditions of the OA, and use of healthcare services. Sample size was calculated using unilateral statistical tests with a 95% confidence level and a power of 80%. Additionally, the sample size calculation accounted for several scenarios based on the effect of the program, including expected effects of 1, 2, 3, 4, 5, 10, 15, and 20 percentage points for each of the 35 indicators proposed. The results of the analysis revealed that a sample size of 1,500 elderly per evaluation group would suffice to detect program effects of up to 4 or 5 percentage points in all of the variables.

The baseline survey was conducted in October-December 2007 among a total of 516 localities in seven states of Mexico. Out of the targeted 6,000 interviews of OA, 5,465 OA (a 91% response rate) were interviewed during baseline. The follow-up survey was conducted in November-December 2008, and it was possible to locate and interview 5,270 OA, or 96% of the OA interviewed at baseline.

**Figure 1:**
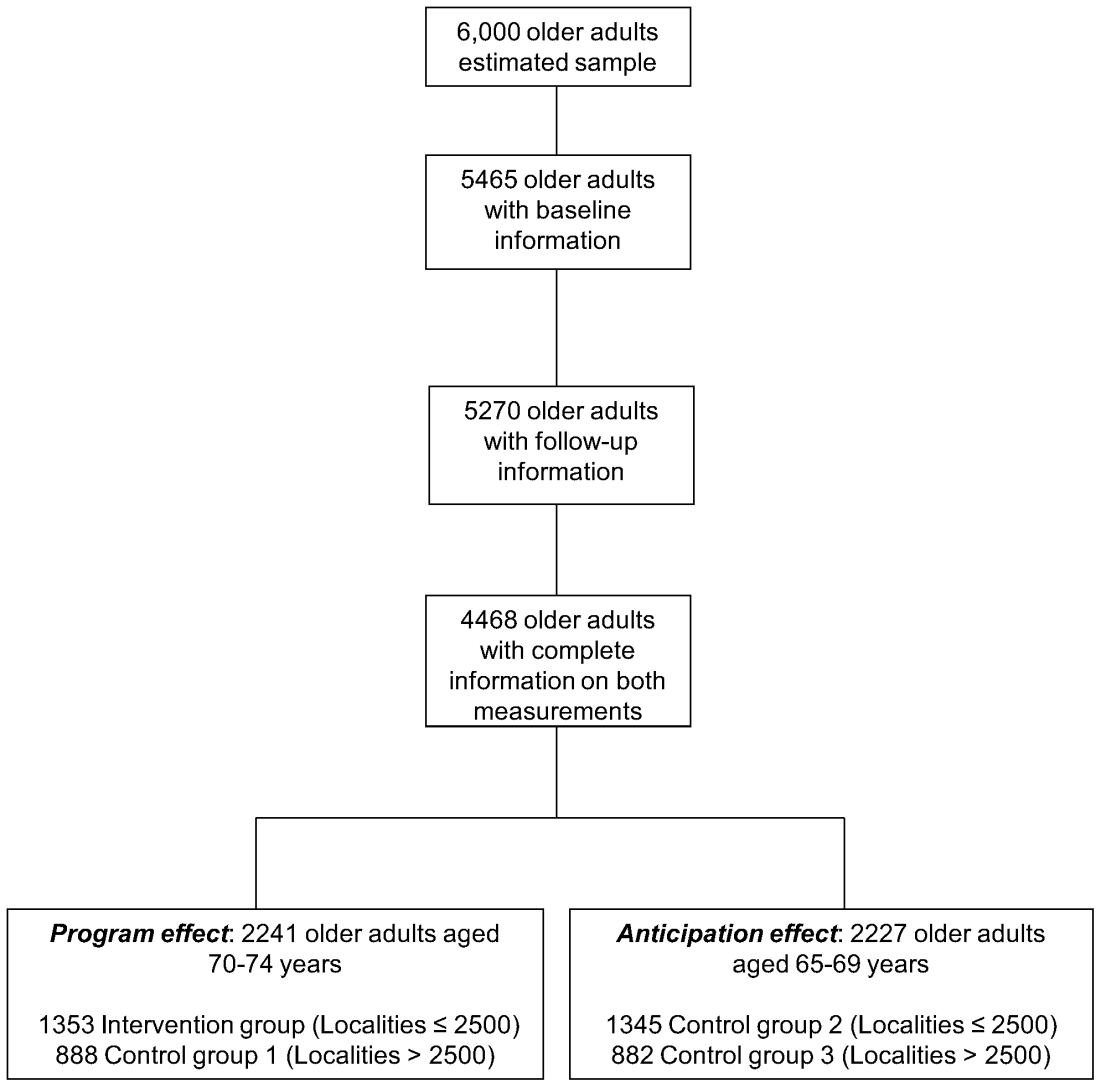
Analytic sample definition.

Inclusion criteria for the present study sample were those subjects with information on both measurements: baseline and follow up. Figure 1 shows the sample included for the impact analysis of the *70 y más* program. As can be seen from the size of the originally estimated sample (6,000 elderly), 5,465 OA were interviewed at baseline, and 5,270 of these were interviewed during follow-up. The final analytical sample was defined based on the common rule from studies on OA that only mentally apt individuals or their caregivers (defined as the people who provide assistance to the OA in their basic and daily activities) can respond to survey questions. By this definition, caregivers can actually answer all questions related to the OA (e.g. demographics, labor, socioeconomic) except for the ones related to perception. For instance, a caregiver should not answer a question which requests information on the OA's emotions. Thus, any survey questions where the OA have to express their feelings or emotions are not answered by caregivers. Thus, of the 5,270 observations existing in both baseline and follow-up (Figure 1), 802 had cognitive impairment in either baseline or follow-up, and including those who had the caregivers respond to all sections with the exception of the sections on mental health, the final sample for the mental health indicators were 4,468 OA.

OA were interviewed at home by standardized personnel working for the National Institute of Public Health. Both at baseline and follow-up, data collected featured the following characteristics: socio-demographic, education, lifestyles, physical and mental health, nutrition and use of health services.

### Measures

As impact indicators of mental well-being we used the following measurements:

#### Depressive symptoms

Within the framework of research on OA depression, the Geriatric Depression Scale is one of the most commonly used instruments. Developed by Sheikh and Yesavage, the scale has been validated in numerous countries and contexts [22], [23]. Currently, it is the most frequently used instrument for measuring depression among the OA, including those residing in poor or marginalized conditions. Depressive symptoms (DS) were assessed using the Geriatric Depression Scale (GDS) 15 items version. We defined a dummy variable equal to 1 if the older adult showed significant DS (GDS ≥6) and 0 otherwise.

#### Empowerment

One of the most immediate and likely effects of the program 7*0 y más* relates to the autonomy and empowerment of the elderly. To incorporate these two dimensions into our evaluation, we applied the World Health Organization (WHO) recommendation on active aging [24]. Specifically, the capacity of OA to participate in household decision making was used as the basic indicator for gauging the extent to which OA were empowered. This gave rise to two indicator variables (dummies): the first was equivalent to one where the older adult declared that he participated in important (non-economic) household decisions; the second was equivalent to one where the older adult declared that he participated in household decisions pertaining to expenses.

#### Statistical analysis

Assuming that our evaluation design succeeded in replicating the environmental conditions of the program and its beneficiaries, it was only necessary to carry out a simple comparison between the average of any indicator of interest for the intervention group and the average of that same indicator for the control group to estimate the program effect. However, this assumption can bias the results greatly, since it is possible that not all observable and unobservable differences between the intervention and control groups will have been removed by our design.

For this reason, we took advantage of the differences-in-differences (DD) model to estimate the program effect. Instead of analyzing the differences between the variables across treatment and control, this model allows us to analyze the differences in change between treatment and control groups by accounting for two types of potential differences between the groups: (1) the differences that existed prior to the intervention (i.e. at baseline or pre-intervention) between treatment and control groups, and (2) the differences arising from unobserved factors at the local level that do not change between baseline and follow-up data collection, which in this case is 2007 and 2008. The DD model is then based on the assumption that in the absence of the program, the change observed in the intervention group would have been the same as the change observed in the control group, or more succinctly, the trends of both groups would be equal. If there were differences between the groups for unobserved characteristics that vary over time and these were associated with program exposure, the DD model would generate biased estimates of the program effect. However, it is expected that the DD model removes a large proportion of the possible causes of bias in its estimates.

The general DD linear probability model for estimating the impact of the program is specified as follows:
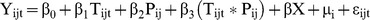
Where Y_ijt_ is an outcome variable for individual i who lives in locality j at time t. T_ijt_ is an indicator variable that takes a value of 1 if the measurement of individual i is in the post-intervention survey (2008) or 0 if it is in the baseline survey (2007). P_ij_ represents an indicator variable that takes the value of 1 if individual i belongs to the intervention group or 0 if he or she belongs to the control group 1, while the term (T_ijt_*P_ij_) represents the estimate of the program impact, X it's a time-varying covariates vector, μ_i_ represents a fixed effect at individual level, and ε_ijt_ is the error term.

The DD model permits the identification of the treatment effect under the assumption that the change in the treated group in the absence of the program would have been the same as the observed change in the control group. When applying this model, the DD model already controls for fixed characteristics over time, thus, only time-varying covariates will be used, both individual/household and locality levels. Differences were considered statistically significant if *p*<0.05, and considered marginally significant if 0.05<*p*<0.10. All analyses were performed using STATA 13.1.

We also conducted additional analyses to complement the DD models (with fixed effects) in combination with propensity score matching as a strategy to verify the robustness of our results, and we verified the assumption of parallelism regarding the DD models. The results of these analyses and their rationale are presented in the Appendix S2 in File S1. Finally, we conducted an alternative analysis to test the robustness of our results, moving the older adults aged 69, and who were in the control group at baseline, to the intervention group.

### Qualitative component

The qualitative study was conducted in January-February 2009 and was based on the data collected during the baseline measurement of the quantitative component. The objectives and research questions were established in accordance with the quantitative component in order to utilize the qualitative exploration to generate a complementary and expanded triangulation of the results.

The qualitative sample was purposively selected with maximum variation criteria to achieve maximum representation of the different subgroups observed [25]. While no established formula exists for defining the number of cases that should be selected for each minimum sample unit, other large-scale evaluation studies [26] have selected a minimum number of three cases per minimal sample unit, to achieve a so-called theoretical or data saturation [27]–[29].

We included OA that have been captured in the quantitative baseline survey. Four localities were selected in two of the seven participating states at baseline. To select the participating states, certain criteria were used on structural characteristics that could determine the experience of the beneficiaries with respect to the program. Selected states shared the same levels of deprivation, migratory rates, and indigenous population proportion. For the selection of localities, the difficulty of accessing health services and the ethnicity composition of its inhabitants were also considered so that the perceived experience of the program could be determined by those characteristics. Sampling scheme for qualitative component can be found in Appendix S1 in File S1.

#### Data collection

Total sample consisted of: 129 semi-structured face to face interviews: 99 program beneficiaries; 16 potential beneficiaries; and 6 suspended OA beneficiaries; 8 interviews with local key actors (two per locality interviewed in public places like parks or schools), and 4 observations from support delivery, one in each selected locality. No refusals were faced during fieldwork.

Interview guide had four sections, self-perception of mental and physical health, use of program's transfer and decision taking over the money, self-perception of social network (family, friends and community) and evaluation of perceived impact of the program. Interview guide was piloted with 5 OA in a non-selected locality.

The fieldwork was conducted in February-April 2009 by a team of five female cultural anthropologists (two of them with M.A.) who lived in the study sites during data collection. In those localities where OA only spoke indigenous language three female indigenous translators helped in order to translate questions and answers for the interviews. All team members were previously trained and have extensively experience doing fieldwork. All interviews and observations were audio-recorded, previous signed of the informed consent, and totally transcribed along with the ethnographic notes, and recorded in field diaries. Notes from the field diaries underwent content analysis [28], [30] together with the interview transcripts in order to achieve data triangulation [30], [31].

#### Analysis

The information generated by the qualitative component of the study consists of ethnographic data composed of selected excerpts from the transcripts collected from semi-structured interviews [32], non-participant observations [33], and fieldwork diaries [28].

The interviews were coded by all team doing fieldwork using pre-defined analytical codes, and live or empirical codes using the program NVivo 2. A content analysis [30] was conducted in order to find meaningful content and recurrent themes in the interviews and observations through deduction and inference [30]. Participants did not provide feedback on the findings. Aside from a preliminary analysis, the content analysis provided a deeper look at the central findings and common themes across one or more groups of individuals, as well as anything that may have reflected an exception or extraordinary perspective, which can help to explain the realities experienced by the individuals interviewed or observed. Major and minor themes where identified, but for the means of this paper, only major or central themes are reported. The validity of the final inferences of the qualitative analysis was confirmed through two types of methodological triangulation of data: (1) data triangulation, due to different voices and tools, and (2) analytical triangulation, result of different social scientists independently analyzing the same ethnographic data [28], [31], [34]. For this analysis, we extracted fragments of the transcripts from participant interviews. Codes used for the present analysis were self-perception of health (physical and mental), perceived health impact, decision making and use of money and perceived impact on social relationships (family and community). The excerpts that sustain the results presented were chosen among others that show similar patterns, because they were particularly emblematic. Hence, those testimonials used to illustrate our findings were chosen, because they articulately describe similar individual, social, and structural characteristics expressed in the interviews that are representative not only of this individual, but also of many other individuals as well.

Additionally, by using triangulation, the internal validity of the data was achieved. By stating the limitations of the qualitative data implicit in the variation of the characteristics of the participants involved in the qualitative exploration (e.g. men and women belonging to indigenous and mixed communities in two states of the country), we sought to create reflexivity to reduce selection bias.

## Results

The quantitative baseline findings can be found in Table 1. For the outcomes variables, significant differences were not observed. In more detail, we observed that prevalence of depressive symptoms were not different across the study groups (25%), being the prevalence of these symptoms similar to the levels found in rural populations. On the other hand, for empowerment indicators, a relatively homogenous distribution was found in the comparison groups, and a high proportion of elderly claim that they contribute to decisions related to spending the household income, as well as to other types of decisions related to household organization. Regarding the covariates (at individual, household and locality levels) we found significant differences in almost of them.

**Table 1:** Baseline characteristics by study group.

	Program effects			Anticipation effects		
	Intervention (70–74 years)	Control group 1 (70–74 years)	p-value^1^	Control group 2 (65–69 years)	Control group 3 (65–69 years)	p-value^1^
	n = 1353	n = 888		n = 1345	n = 882	
**Outcomes**						
Depressive symptoms	0.25 [0.01]	0.25 [0.01]	0.655	0.24 [0.01]	0.28 [0.02]	0.042
OA participates in household decisions	0.72 [0.01]	0.74 [0.01]	0.268	0.76 [0.01]	0.80 [0.01]	0.025
OA participates in household spending decisions	0.69 [0.01]	0.72 [0.02]	0.201	0.74 [0.01]	0.76 [0.01]	0.143
**Individual covariates**						
***Time-stationary***						
Sex (female)	0.50 [0.01]	0.64 [0.02]	<0.001	0.43 [0.01]	0.46 [0.02]	0.206
Literacy	0.33 [0.01]	0.37 [0.02]	0.047	0.36 [0.01]	0.41 [0.02]	0.019
Indigenous	0.35 [0.01]	0.30 [0.02]	0.018	0.37 [0.01]	0.33 [0.02]	0.052
***Time-varying***						
Age	72.52 [0.04]	72.57 [0.05]	0.446	67.43 [0.04]	67.54 [0.05]	0.078
OA living alone	0.04 [0.01]	0.13 [0.01]	<0.001	0.03 [0.00]	0.09 [0.01]	<0.001
OA having paid job	0.36 [0.01]	0.31 [0.02]	0.007	0.48 [0.01]	0.47 [0.02]	0.930
OA head of household	0.65 [0.01]	0.73 [0.01]	<0.001	0.72 [0.01]	0.79 [0.01]	<0.001
Number of co-moribidities^2^	0.84 [0.03]	0.89 [0.04]	0.300	0.72 [0.03]	0.73 [0.03]	0.845
Functional dependence	0.28 [0.01]	0.30 [0.02]	0.304	0.24 [0.01]	0.25 [0.01]	0.677
Marital status (married/cohabitating)	0.65 [0.01]	0.46[0.02]	<0.001	0.67 [0.01]	0.60 [0.02]	0.001
**Household covariates**						
Household size (# of equivalent adults)	5.16 [0.08]	3.67 [0.09]	<0.001	5.56 [0.08]	4.22 [0.09]	<0.001
Asset index	0.19 [0.03]	−0.14 [0.04]	<0.001	0.13 [0.03]	−0.17 [0.04]	<0.001
Enrolled in Oportunidades program	0.68 [0.01]	0.76 [0.02]	<0.001	0.68 [0.01]	0.82 [0.01]	<0.001
**Locality covariates**						
Financial services (Bank or saving popular services)	0.07 [0.01]	0.30 [0.02]	<0.001	0.07 [0.01]	0.30 [0.02]	<0.001
Basic services (electricity, water, sewer, garbage collection)	0.79 [0.01]	0.76 [0.02]	0.170	0.79 [0.01]	0.76 [0.02]	0.170
Educational infrastructure (primary and secondary schools)	0.03 [0.01]	0.37 [0.02]	<0.001	0.03 [0.01]	0.37 [0.02]	<0.001
Health services (any hospital, clinic or office doctor)	0.82 [0.01]	0.91 [0.01]	<0.001	0.82 [0.01]	0.91[0.01]	<0.001
Basic trade services (selling food and household goods)	0.69 [0.01]	0.79 [0.01]	<0.001	0.69 [0.01]	0.79[0.01]	<0.001
Basic communication services (telephone or telegraph)	0.41 [0.01]	0.65 [0.02]	<0.001	0.41[0.01]	0.65 [0.02]	<0.001
Any incident in the locality in the last four years (droughts, floods, frosts, fires, plagues, earthquakes, hurricanes)	0.61 [0.01]	0.54 [0.02]	0.002	0.61[0.01]	0.54 [0.02]	0.002

OA: Older adult.

Standard error in brackets.

1 p-value for a t-test or z-proportion test.

2 Hypertension, diabetes, dyslipidemia, myocardial infarction, angina pectoris, heart disease, stroke, chronic lung disease, osteoporosis, and cancer.

The findings for the depressive symptoms indicator show the program had a significant overall effect on them. The negative value of the associated coefficient (−0.063, p<0.05, Table 2) reflect the fact that the program contributes primarily to greater feelings of safety and welfare associated with decreased depressive symptoms among the OA.

**Table 2:** Overall effect on mental well-being indicators: Depressive symptoms and empowerment.

Effect	Depressive symptoms (GDS≥6)	Participates in making household decisions	Participates in household spending decisions
Intervention	−0.063** [0.031]	0.097*** [0.031]	0.116*** [0.033]
Anticipation	0.053* [0.029]	0.047 [0.029]	0.076*** [0.028]
**Sensitivity analysis**			
Intervention£	−0.057** [0.028]	0.085*** [0.030]	0.095*** [0.032]

£ Moving older adults (aged 69) from control group at baseline to the intervention group.

GDS: Geriatric Depression Scale.

Linear probability models with fixed effect at individual level, adjusted for time-varying covariates in Table 1.

Standard errors in brackets.

*p<0.10; **p<0.05; *** p<0.01.

We also found a strong trend, shared by the vast majority of participants, regarding the reduction of sadness, and feeling of increasing empowerment. According to their words OA experienced a reduction or a relief of poverty and the stress related with having no income at all for most and an increased sense of security and well-being from receiving a regular income that they could consider their own and on which they can decide what to do with. In the following testimonials, we can see how a non-indigenous woman expresses her sadness as 'shame' when not having money and when being sick and having to look for money within her networks. In the second testimony below, we see how an indigenous man declares being happy, since he has something to eat.

Q. …and about the program, how do you feel? Do you feel that your health has changed anything since you had the program?

*R. Now I eat better, now I have ‘a cent’ to buy at least a piece of meat, something like that… some bread. Before it was not like this, we couldn't buy anything because we did not have money (laughter), and now yes, now we have this. I do feel better.*

Q. And about how you feel, do you feel any change?

*R. No, I feel happy (laughter). I do, maybe there is a person who is not happy, but I do feel happy.*

Q. And do you think that how you feel is related somehow to the program, or is it because you are indeed a happy kind of person anyway?

*R. Well, indeed I did not feel sad, I was ashamed because the money was not enough to buy needed things, and then, when I got sick, I used to have to beg for money. But now, at least we have some money. If I get sick at least I have to buy medicine that [the health services] don't have. (SanBe, Non-indigenous man)*

On the other hand, the indigenous man expresses his joy at receiving the program:

Q. Please ask him how he feels now that he is receiving the program? How does he feel about getting [the support], does he feel comfortable, quiet, or does he feel bad or stressed, how does he feel?

*Translator R. (Laughter) He says that when he receives the support he feels happy and brings some meat and more things. When he is home, he meets my godmother [elder's wife], close together, happy because they receive the support.*

Q. And now, finally I only wanted to ask if he feels that after receiving this money has something changed in his health and mood?

*Translator R. He feels better because before that support, he had no support, but since he already has support he feels better, more comfortable. But before, he says he did not, because sometimes he had no money to buy a little something for the kitchen. Well now he feels better because he already has the support. (Ahitic, Indigenous man)*

Many of OA declared that pension was their only source of income at the moment. Moreover, because of the conditions in rural areas, which can also be characteristically peasantry, where most of the beneficiaries had lived their entire lives, receiving a stable and fixed monetary income was declared by OA, especially women, to be an event which had never before been experienced. This suggests that there was a significant perceived impact for these beneficiaries.

In empowerment, the overall effect of the program was significant for: *participates in household decision making* (p<0.01, Table 2) and *makes decisions on household spending* (p<0.01, Table 2). Specifically, receiving a pension boosted the percentage of OA participating in decision making at home by 9%. Likewise, the percentage of OA participating in decisions regarding household expenses rose by 10% in the intervention group. Results therefore indicate that, in general, the Program exerted a significant impact on the empowerment level of *70 y más* beneficiary OA.

Sharing their money with their household constituted an important action for the beneficiaries because by contributing for the household income they felt empower to give their opinion regarding household's decision. Thus, it can be inferred from the transcripts of the OA, that the practices and their significance in the communities can explain and make sense of the context at which we have arrived through our statistical findings on mental health and empowerment. The elderly consistently claimed that the money was not taken away by their families and that they themselves decided how and on what to use it. Several speeches support the fact that OA decide to share their money with their families and contribute as well to the wellbeing of their households. Here is a non-indigenous man talking about it:

Q. So, for example, do [your daughters] tell you how to spend your money?

*R. No, no, nothing like that. They don't even ask me or tell me what to do with my money… I know I have to help them. But they don't take away anything from me, I have the money, I keep it. Anyway I give them money because I know they don't have, because they are poor and they don't have enough money for medicine for example, neither for transport.*

Q. Ok…

*R. But they are not telling me what to do, they don't tell me ‘now you have money give it to us’, no. It is clear, the money is mine and I do decide about it. (SanBe, Non-indigenous man)*

Having one's own resources also gives a sense of economic independence for OA who in many cases are financially dependent on their children, whose own families demand the majority of their resources. For many women, the money they receive through the program *70 y más* is the first economic transaction of their entire lives, which has very revealing impact implications on autonomy, and empowerment. These women expressed in the semi structured interviews how the money gave both parts, themselves and their children, a kind of relief, feeling better for not only reduce the economic burden in their children, but gaining independence in terms of how to spend their own money.

In these interviews a man and a woman express their economic independence from their networks and children.

*R. I, what I have left, I keep it [for savings] and… when I'm sick, well, not telling anyone to borrow me money. (SanBe, Non-indigenous woman)*

Q. and how do you feel now that you have the Program?

*R. Oh! It is a great help, because I don't have to ask my children anymore for help.*

Q. And about them, how do you feel they feel about this?

*R. I believe that they are ok with this. I say it because they do not worry anymore. They are not thinking that they have to help me. (SanBe, Non-indigenous man)*

So then, by being able to offer to their household financial support to purchase food, OA creates reciprocity among they and their families obtaining acknowledgement from the household members and are empowering themselves to decide on issues that concern their lives, as well as the collective life of the household. In general, the diverse elements described around the noncontributory pensions contribute in different ways through complex social mechanisms to '*reduce suffering*', '*give themselves value*', '*increase happiness*', as well as to improve the emotional health.

In the narrative of this indigenous woman reciprocity and empowerment are feelings expressed in relation to the program:

*T. Mmm… She says that she feels good with the 70 y más program. She feels that it has helped her. She says she feels more important with this money, mainly in relation to her family because with this money she can help her daughter a little bit. She also says that she helps her daughter in embroideries; when her daughter cannot finish them, she helps her.*

Q. And could you ask her what the most important thing is that she feels she gives her family?

*T. She says that she cannot give them more than sharing her food. She says she always cooks for her grandchildren because they help her, too. When [her grandchildren] come, they give her some 1000 pesos, or they bring some [soda beverage] or some clothes. She says that it is because that she always prepares some food for them. That is all of what she gives them. (Ahitic, Indigenous woman)*

### Anticipation Effects

A possible effect of the program *70 y más*, given its eligibility criteria, is that OA a little younger than 70 years old and residing in localities with less than 2,500 inhabitants could modify some of their habits and behaviors related to income, and at the same time, they may be able to modify some dietary habits or some of their social, family-related, or personal expectations, particularly in terms of their emotional health. Although it has been referred to as different names in the literature, this process can be designated as the anticipation effect.

An advantage of our evaluation design is that we can identify the presence, or absence, of a potential anticipation effect and its magnitude. These reflect not only the extent the program would have on impacting future beneficiaries, but also a generalized idea of the real magnitude of the program effect. In order to estimate the anticipation effect, the same specification of our models described earlier was used with the inclusion of OA aged 65 to 69 years old from control groups 2 and 3. Table 1 shows the descriptive results comparing both groups with significant difference for almost all covariates.

For depressive symptoms (Table 2) we observed a significant effect of the program, with a negative coefficient associated (implying a decrease in depressive symptoms) and a positive coefficient for the anticipation effect, which means that in absence of the program there must be an increase of depressive symptoms, which in turn indicate that the estimated program effect could be even larger. As for the empowerment indicators (Table 2), significant effects (with positive coefficients) of both the program and anticipation were detected. For all these indicators, the magnitude of the anticipation effect is lower compared with the program effect.

### Sensitivity analysis

Results of the analysis that included OA aged 69 (and who were in the control group at baseline) in the intervention group are in the sensitivity analysis panel of Table 2. As can be seen, the program effect remains significant for all indicators analyzed, although its magnitude is slightly smaller.

For the mental well-being outcomes using DD model estimates combined with the propensity score matching algorithms the results can be found in Table 3. Results obtained with the DD model were not modified by the matching models, with similar levels of significance and magnitude of the coefficients. For the analysis to test the parallelism assumption of DD models, results from comparing the alternative control groups appear to support the assumption of parallelism since just one coefficient is significant, whilst in the analyses with a series of alternative indicators was observed that the coefficients are statistically equal to zero, suggestive of evidence in favor of the assumption for parallelism (Tables S4 and S5 in File S1).

**Table 3:** Overall effect on mental health indicators.

	Depressive symptoms (GDS≥6)	Participates in making household decisions	Participates in household spending decisions
All sample	−0.063**[0.031]	0.097*** [0.031]	0.116*** [0.033]
Matched sample			
Caliper^1^	−0.067* [0.034]	0.110*** [0.035]	0.119*** [0.037]
Kernel-based^2^	−0.062**[0.031]	0.101*** [0.032]	0.110*** [0.033]

Alternative strategies of estimation

GDS: Geriatric Depression Scale.

1 Caliper algorithm with a specified distance of 0.0005 and one-to-one merge (713 units in intervention and control groups).

2 Using epanechnikov kernel, and one-to-one matching (875 units in intervention and control groups).

Linear probability models with fixed effect at individual level, adjusted for time-varying covariates in Table 1.

Standard errors in brackets.

*p<0.10; **p<0.05; *** p<0.01.

## Discussion

In our analysis context, we hypothesized that the program *70 y más* could have an impact, related to the economic transfer, on the analyzed mental well-being indicators, because the transfer can be seen as a component of the SES among OA beneficiaries. The most conclusive result found in our analyses was the effect of the program on the presence of depressive symptoms, which can be interpreted from the OA transcripts. This is a very important finding, since of all mental health problems experienced by OA, the most important based on prevalence is depression. This issue is even more pressing because depression is associated with increased mortality and suicide, as well as morbidity, in terms of functional dependence [35]–[39]. So, if program has a significant effect on depressive symptoms, it is possible that it will also have an indirect effect on the mortality and disability of OA, an effect that can only be measured in the long term.

This same argument could be generalized as follows. If non-contributory pensions have a positive effect on economic and well-being indicators [11], [15], [16], then it is possible that also contribute to a healthy and active aging [24], which in turn will impact on the health status of older adults, and will decrease disability and mortality in this age group.

A significant effect was also observed for the OA empowerment, measured through the decision making at home and decision making on household expenditure, which can be found in the literature related to empowerment [40], [41]. It is important to note that the effect seems to be more important for women (taking into account the qualitative testimonials), perhaps because for many of them this is the first time they have their own income, which could particularly favored a strong empowerment process. In addition women are more often widows, so paying third parts for work can make them a stronger feeling of empowerment and decision making.

Following a constructivist interpretative framework [42], understood as the collective generation of reality and transmission of modulating experiences, it can be deduced from the qualitative data that when receiving the pension, OA perceive the amount of money as their own and that it comes constantly (e.g. they know it reliably comes every two months), and this perception results in a feeling of a reduction of stress or sadness normally caused by poverty and uncertainty or total lack of income. This reduction of stress is meant to be a feeling of safety and welfare.

The interviews with OA help us to understand the mechanisms behind income appropriation and decision making within the household. Furthermore, the results presented here indicate that social mechanisms are at play under which the beneficiaries experience an effect from the pension that redefines the meaning of money in their lives. Thus, besides meaning a perceived reduction of poverty, the pension the OA receive has a positive effect on self-determination and decision making at both the individual and household levels. This decision-making power is translated into economic autonomy, self-sense of value, feeling of reciprocity and worth and the reduction of economic dependence on their children.

In turn, OA share or redistribute the income they receive across their household as found in the study by Marquez-Serrano [43]. In doing so, they regain the power to give their opinion on household matters, which in turn revitalizes their social networks through reciprocity and the sense of being recognized and not being a burden for their families. The resulting effects of these feelings have been previously identified in the literature on OA social networks [44]–[48], and are expressed by the OA participating in the present evaluation as '*no longer suffering*', '*feeling valuable*', and '*being happy*'.

Despite the beneficial aspect that is associated with increasing levels of decision making, it is also possible that there is a downside. For example, it is possible that older adults perceive the decision as a burden, as they lack strong social networks. While this is something that could not be analyzed in this study, it is important that future research will address this issue.

A central finding of the study was related to the use of the pension. The fact that OA make their own decisions on how and on what to spend their money is a key element linked to the feelings of empowerment described earlier. Decision making and the effects of decisions make up the key mechanisms that largely explain the feelings of reduced sadness, which can be associated with depressive symptoms.

Regarding anticipation effects it can be argued that since empowerment is associated with a higher income among OA, it is possible that in households where an OA is about to receive the *70 y más* program pension, the family dynamics can begin to change towards greater respect and consideration for the potential OA beneficiary, because they anticipate an economic transfer and along with it, potential economic benefits for all household members derived from the OA's decision to share their new income. However, it is important to emphasize that family dynamics may also be modified in an undesired manner. For example, it is possible that household members starting to treat the OA better knowing that in the short term could take the pension money. This is an issue that also must be analyzed in future research.

Beyond this discussion, presence of anticipation effects implies that the program's impact would be even greater, since the absence of expectations of becoming a *70 y más* beneficiary would not have had a modification to empowerment. In fact, and in our estimates, the difference observed between beneficiaries and non-beneficiaries would be larger.

Using matching techniques in our analysis confirms the robustness of our results regarding the significant effect that the *70 y más* program has on various indicators of mental well-being in OA residing in rural areas of Mexico.

We have mentioned that a great body of evidence on non-contributory pension's impact has mainly focused on socio economic indicators. This is an important flaw because research indicates that in old age health mental problems are highly prevalent among OA. Due to its devastating consequences, mental health represents an important public health issue [49].

Even so, some studies have analyzed the effect of non-contributory pension on OA's health status. A first set of studies demonstrated that the South African pension improved the self-report health status of its beneficiaries and the co-residing members of the household [50], whereas other showed that pensions are associated with the social well-being and quality of life [17], [19]. In other study, using a quasi-experimental design, was evaluated the short-term effect (6 months) of a non-contributory social pension introduced in urban localities in Mexican State of Yucatan on a range of indicators including health. Despite of having found significant effects on labor supply, food availability, medical consumption, and memory, they did not find effect on depressed mood (dysphoria) [51].

Several limitations can be noted in our study. First, given we used a discontinuity regression approach to form our comparison groups, our estimate of the *70 y más* impact is a local estimator; this means that the observed effects are valid only for OA aged 70–74. So it is still pending analyze the impact of the program in older ages. Second, since *70 y más* started in rural localities, our analytical sample is also restricted to rural areas of Mexico, so nothing is known about the program's effect in urban areas where the program also operates nowadays. Third, we just had a short time of the exposure to pension (11 or 12 months), so our impact estimate reflects only an immediate effect, and it is important to determine the medium and long terms effects of the program. And fourth, the qualitative component of the study does not have a baseline measure and only took into account OA beneficiaries of *70 y más*. Even and when there is a debate about the relevance of using a control group from a constructivist point of view, the study could be have more robust results if qualitative component had been included a control group and/or a baseline measurement.

Finally, some of our results showed that if an intervention were implemented to increase the income and the SES of OA, some mental health outcomes may change, just as proposed in the literature [52], [53]. For now, and in the short term, it is reasonable to think that this effect is almost exclusively attributable to the economic transfer and the strong sense of solidarity and sharing behind the social mechanisms described that could be observed in the short term through the qualitative component. Perhaps in the medium and long terms, it will be possible to identify other psychosocial or behavioral factors that contribute to explaining the effects observed.

## Supporting Information

File S1 **This file contains Appendix S1, Appendix S2, Table S1, Table S2, Table S3, Table S4 and Table S5.** Appendix S1, Intervention and control group selection and Sampling scheme for qualitative component. Appendix S2, Propensity score matching and Parallelism assumption of DD model. Table S1, Probit regression to predict enrollment in *70 y más* program. Table S2, Results of matching using caliper algorithm. Table S3, Results of matching using kernel algorithm. Table S4, Testing the parallelism assumption: alternative control groups. Table S5, Testing the parallelism assumption: alternative outcomes.
(DOCX)
