# Supplementary material for: Impact of the Non-Contributory Social Pension Program 70 y más on Older Adults’ Mental Well-Being
Source: PLoS One. 2014 Nov 19;9(11):e113085. doi: 10.1371/journal.pone.0113085 (PMC4237374; doi:10.1371/journal.pone.0113085)
Supplement: File S1 — This file contains Appendix S1, Appendix S2, Table S1, Table S2, Table S3, Table S4 and Table S5. Appendix S1, Intervention and control group selection and Sampling scheme for qualitative component. Appendix S2, Propensity score matching and Parallelism assumption of DD model. Table S1, Probit regression to predict enrollment in 70 y más program. Table S2, Results of matching using caliper algorithm. Table S3, Results of matching using kernel algorithm. Table S4, Testing the parallelism assumption: alternative control groups. Table S5, Testing the parallelism assumption: alternative outcomes. (DOCX) [file pone.0113085.s001.docx]

**Appendix S1**

*Intervention and control group selection*

The variable representing age determined treatment assignment, with 70 years defined as the cutoff point. The intervention group was defined as OA aged 70-74, and a first control group as elderly aged 65-69 living in the same communities. The range of the age window was determined by an analysis of 127 household and individual level indicators from three national surveys in Mexico: (a) the 2006 Seguro Popular Universal Health Insurance Impact Evaluation Survey [1]; (b) the 2002 National Performance Evaluation Survey [2], and (c) the 2001 Mexican Health and Aging Study [3]. We compared the indicators for the different aforementioned age ranges on both sides of the cutoff point and confirmed that the widest age window reflecting homogenous groups on both sides of the cutoff was composed of age groups 65-69 and 70-74 (i.e. these groups were homogeneous on the largest majority of characteristics: 112 out of 127).

Because in 2007 the program selectively operated only in localities with 2,500 or fewer inhabitants, we used the population size of each locality as a second criterion to select additional control groups, using the population size of 2,500 inhabitants as the cutoff point. The second and third control groups were selected from communities with 2,501-2,700 inhabitants. This range of community size was determined by an analysis of 41 locality level indicators from *Conteo 2005*, a large inter-Census household survey conducted by the National Institute of Geography and Informatics in Mexico in 2005 [4]. After trying different locality size ranges, we verified that the localities on both sides of the 2,500 cutoff (i.e. with less than 2,500 and with 2,501-2,700 inhabitants) were homogenous according to a large majority of indicators (35 out of 41).

In regard to economy, well-being and health status, *70 y más* may also be motivating changes in the practices and activities of OA under 70 years of age and living in the same beneficiary communities. We hypothesized that knowing that a monetary benefit is forthcoming at age 70 might trigger changes in spending and consumption patterns as well as health conditions as individuals anticipate a more favorable life afforded by the program. If so, 65-69 year olds in the beneficiary communities may change their practices and activities as a result of the existing program, and thus potentially jeopardize the validity of the first control group. Therefore, a third control group was designed to encompass OA within the same age group (65-69 years) but residing in localities where the program is not in operation. This group will also allow us to estimate the potential effect of program anticipation, a factor that should be taken into account for a global assessment of the impact of the *70 y más* program. This process to identify the intervention and control groups, using a quantitative variable and associated cutoffs is similar to a regression discontinuity design, although in our case was only used for the definition of our four groups.

*Sampling scheme for qualitative component*

Based on the quantitative sample, we took a sample of four localities with comparable levels of development and migration and with the following characteristics: which were not exclusive: indigenous versus non-indigenous population and being close versus far from a health clinic. The criterion related to the distance from a health clinic was established based on medical mapping developed by the Mexican Geographic Information Systems for Health as well as data from the local sanitary authorities. A close distance was defined in relation to whether primary healthcare was situated up to 30 minutes away from the community by public transportation, with a distance of 31 minutes and more considered as far. Thus, the sample was organized to contain two indigenous localities and two non-indigenous localities, and among these four localities, two near a health clinic, and two far from one.

Seeking maximum variation, OA were included if they met certain individual criteria (e.g. sex and health status), social criteria (e.g. having or not having social networks), and criteria reflecting the community (e.g. ethnicity and being near health services) [5]. To gain a better understanding of the subjects and to further understand the perceived impact of the program, several other key actors of relevance were included to reflect the viewpoints of potential beneficiaries and OA with physical disabilities [6]. Additionally, nonparticipant observations were carried out at the location of payments on the day the program handed out pensions in each of the localities to observe the conditions under which support is delivered to the OA and to observe the dynamics between program implementers and beneficiaries. The final sample for the qualitative study included four different types of actors: (1) OA beneficiaries (2) OA potential beneficiaries^[[1]](#footnote-1)^, (3) suspended OA beneficiaries^[[2]](#footnote-2)^, and (4) key actors^[[3]](#footnote-3)^.

**References**

1. SSA (2006) Seguro Popular: Encuesta de Evaluación de Impacto. Dirección General de Evaluación del Desempeño: Secretaría de Salud (SSA), Gobierno de México. Retrieved from: http://www.salud.gob.mx/unidades/evaluacion/seguropopular/seguropopular.htm.

2. SSA (2002) Evaluación del Desempeño de los Sistemas de Salud. Dirección General de Evaluación del Desempeño: Secretaría de Salud (SSA), Gobierno de México. Retrieved from: http://www.salud.gob.mx/unidades/evaluacion/evaluacion/evaluacion.htm.

3. Wong R, Espinoza M, Palloni A (2007) [Mexican older adults with a wide socioeconomic perspective: health and aging]. Salud Publica Mex 49 Suppl 4: S436-447.

4. INEGI (2005) Conteo de Población y Vivienda 2005: Instituto Nacional de Estadística y Geografía, México. Retrieved from: http://www.inegi.org.mx/est/contenidos/proyectos/ccpv/cpv2005/default.aspx.

5. Teddlie C, Yu F (2007) Mixed Methods Sampling: A Typology With Examples. Journal of Mixed Methods Research 1: 77-100.

6. Connell Szasz M (2001) Between Indian and White Worlds. The Cultural Broker. Norman, Oklahoma: Univ of Oklahoma Press.

**Appendix S2**

*Propensity score matching*

As observed in the findings from baseline comparisons across groups of interest (adults aged 70-74, with and without intervention; Table 1), significant differences were found in various observed characteristics. This was one of the main reasons for selecting the DD model. However, because the groups were not well balanced at baseline, the results could be biased in some way. This could be mainly because the small localities (< 2500 inhabitants) may have distinct characteristics regarding larger localities, for example, the access to health services. So, in order to check the robustness of our results, we have carried out alternative analyzes using the propensity score matching technique [1] in combination with the differences-in-differences method. For these analyzes we used the intervention group (OA aged 70-74 and living in rural areas) and control group 2 (OA aged 70-74, in localities with 2,501-2,700 inhabitants).

We first constructed a propensity score that estimated the probability of receive the benefits of *70 y más* program given a set of predictors, and we then created a control group (not-enrolled) and a treatment group (enrolled) having similar propensity scores. We used a probit regression model to estimate the conditional probability of *70 y más* enrollment given a set of covariates, and then caliper and kernel-based matching algorithms allowed us to match, one-to-one, enrolled and not-enrolled OA with similar propensity scores [2,3].

To ensure comparability, we tested the balancing property on pre-treatment covariates between *70 y más* enrollees and people not enrolled in the program. We followed the algorithm suggested by Dehejia and Wahba to find the best model specification [4,5]. The method involved the use of different specifications until we obtained a balanced distribution of the following covariates at the individual, household and locality levels: sex, indigenous condition, head of the household, difficulties with basic activities of daily life, difficulties with instrumental activities of daily life, presence of a chronic condition, paid job, OA living alone, civil status, unigeneracional household (just older adults living at home), OA has someone who can be his/her economic support, OA has a house, OA has a car, literacy, total monthly household expenditure, and locality deprivation index. Furthermore, we estimated the percentage bias reduction by calculating the difference in absolute bias between treated and control groups as a percentage of the square root of the average of the sample variances [6].

Results

Table S1 shows the results of the probit regression model to predict the affiliation to the program. In the model, being female, indigenous, or head of the household, and having a paid job, reduces the probability of being enrolled in *70 y más*. Meanwhile, if the older adult knows read and write, and lives in more deprived areas, has more likelihood to be enrolled in *70 y más*.

Matching

Once we estimated the propensity score, we used two different algorithms to match one OA in the intervention group with one OA in control group 2. The results of the matching process were rigorously evaluated to ensure homogeneity in the observed characteristics, except, of course, regarding program participation. In the matched samples, and for both algorithms, the differences between both groups were considerably smaller for most of the variables. Table S2 shows the matching results using the caliper algorithm with a specified distance of 0.0005 and a random ordering of observations. 1426 OA were matched (713 in each group) and there were no significant differences for all variables included in the analysis. In Table S3 are the results for kernel algorithm, for this algorithm 1750 OA were matched, 875 in each group. Also, there no were significant differences in the set of covariates.

*Parallelism assumption of DD model*

Following the recommendations made by Duflo [7], we adjusted the DD models using a pair of alternative control groups (taking advantage of our evaluation design, we account for two control groups of elderly aged 65-59 years), as well as a series of DD models using the original control group (elderly of 70-74 years of age) but with a set of indicators that were not affected by the program.

The results from comparing the alternative control groups can be found in Table S4. These results appear to support the assumption of parallelism, since just one coefficient is significant. Table S5 contains the analyses with a series of alternative indicators. In all the analyses, it was observed that the coefficients are statistically equal to zero, suggestive of evidence in favor of the assumption for parallelism.

Despite what has been mentioned above and in general, the results do not provide categorical or absolute evidence in favor or against the assumption of parallelism. In principle, using alternative control groups of elderly 65-69 years of age probably were not the best option for controls. In fact, it is for the same age effect, that these groups were not used to estimate the impact of 70 y más. Because of that, we are not confident that their use is best suited to test the assumption of parallelism.

On the other hand, the alternative indicators that we have used appear to offer strong evidence in favor of parallelism, because for some indicators, we used the remaining members of the household and not the OA. However, we understand that some of the indicators that we have used could still be questioned with respect to the impact that the program could have on them.

**References**

1. Abadie A, Imbens G (2006) Large sample properties of matching estimators for average treatment effects. Econometrica 74: 235–267.

2. Becker S, Ichino A (2002) The estimation of average treatment effects based on propensity scores. Stata J 2: 358–377.

3. Leuven E, Sianesi B Psmatch2: Stata module to perform full Mahalanobis and propensity score matching, common support graphing, and covariate imbalance testing. Available: http://ideas.repec.org/c/boc/bocode/s432001.html.

4. Dehejia R, Wahba S (1999) Causal effects in nonexperimental studies: re-evaluating the evaluation of training programs. J Am Stat Assoc 94: 1053-1062.

5. Dehejia R, Wahba S (2002) Propensity score-matching methods for nonexperimental causal studies. Rev Econ Stat 84: 151–161.

6. Rosenbaum P RD (1985) Constructing a control group using multivariate matched sampling methods that incorporate the propensity score. Am Stat 39: 33-38.

7. Duflo, E., “Empirical Methods”, MIT Handout, Fall 2002

**Table S1. Probit regression to predict enrollment in *70 y más* program**

|  | Coefficient | Std. Err. | p-value |
| --- | --- | --- | --- |
| Female | -0.368 | 0.081 | <0.001 |
| Indigenous condition | -0.402 | 0.066 | <0.001 |
| Head of the household | -0.444 | 0.089 | <0.001 |
| Difficulties with basic activities of daily life | -0.110 | 0.083 | 0.187 |
| Difficulties with instrumental activities of daily life | -0.034 | 0.093 | 0.716 |
| Presence of a chronic condition | 0.073 | 0.060 | 0.218 |
| Paid job | -0.168 | 0.075 | 0.025 |
| Older adult living alone | -0.464 | 0.116 | <0.01 |
| Civil status | 0.154 | 0.070 | 0.028 |
| Unigeneracional household (just older adults living at home) | -0.221 | 0.070 | 0.001 |
| Older adult has someone who can be his/her economic support | -0.037 | 0.063 | 0.556 |
| Older adult has a house | 0.145 | 0.079 | 0.068 |
| Older adult has a car | 0.130 | 0.078 | 0.094 |
| Literacy | 0.179 | 0.065 | 0.006 |
| Total monthly household expenditure (reference 1st quartile) |  |  |  |
| 2nd quartile | -0.079 | 0.081 | 0.331 |
| 3rd quartile | 0.058 | 0.085 | 0.494 |
| 4th quartile | -0.229 | 0.084 | 0.007 |
| Locality deprivation index | 0.643 | 0.056 | <0.001 |
| Constant | 1.030 | 0.145 | <0.001 |

**Table S2. Results of matching using caliper algorithm**

|  | Matched | Treated | Control | Percentage of bias | Percentage of reduction on \|bias\| | p-value |
| --- | --- | --- | --- | --- | --- | --- |
| Female | Unmatched | 0.50 | 0.64 | -28.6 |  | <0.001 |
|  | Matched | 0.59 | 0.57 | 5.5 | 80.9 | 0.334 |
|  |  |  |  |  |  |  |
| Indigenous condition | Unmatched | 0.33 | 0.37 | -8.7 |  | 0.046 |
|  | Matched | 0.34 | 0.37 | -5.6 | 35.8 | 0.319 |
|  |  |  |  |  |  |  |
| Head of the household | Unmatched | 0.64 | 0.72 | -17.3 |  | <0.001 |
|  | Matched | 0.70 | 0.68 | 4.4 | 74.5 | 0.432 |
|  |  |  |  |  |  |  |
| Difficulties with basic activities of daily life | Unmatched | 0.18 | 0.20 | -5.8 |  | 0.175 |
|  | Matched | 0.17 | 0.20 | -7.2 | -23.4 | 0.191 |
|  |  |  |  |  |  |  |
| Difficulties with instrumental activities of daily life | Unmatched | 0.14 | 0.16 | -6.0 |  | 0.163 |
|  | Matched | 0.14 | 0.16 | -5.7 | 5.1 | 0.306 |
|  |  |  |  |  |  |  |
| Presence of a chronic condition | Unmatched | 0.50 | 0.53 | -4.8 |  | 0.265 |
|  | Matched | 0.52 | 0.52 | 0.0 | 100 | 1.000 |
|  |  |  |  |  |  |  |
| Paid job | Unmatched | 0.21 | 0.22 | -1.6 |  | 0.705 |
|  | Matched | 0.23 | 0.21 | 3.8 | -135.1 | 0.499 |
|  |  |  |  |  |  |  |
| Older adult living alone | Unmatched | 0.04 | 0.15 | -36.6 |  | <0.001 |
|  | Matched | 0.08 | 0.07 | 1.6 | 95.5 | 0.752 |
|  |  |  |  |  |  |  |
| Civil status | Unmatched | 0.65 | 0.46 | 38.7 |  | <0.001 |
|  | Matched | 0.51 | 0.56 | -11.3 | 70.8 | 0.049 |
|  |  |  |  |  |  |  |
| Unigeneracional household (just older adults living at home) | Unmatched | 0.21 | 0.35 | -30.4 |  | <0.001 |
|  | Matched | 0.29 | 0.27 | 2.8 | 90.6 | 0.617 |
|  |  |  |  |  |  |  |
| Older adult has someone who can be his/her economic support | Unmatched | 0.70 | 0.71 | -2.5 |  | 0.566 |
|  | Matched | 0.72 | 0.70 | 5.2 | -107.9 | 0.354 |
|  |  |  |  |  |  |  |
| Older adult has a house | Unmatched | 0.62 | 0.65 | -6.6 |  | 0.130 |
|  | Matched | 0.65 | 0.65 | 0.3 | 95 | 0.953 |
|  |  |  |  |  |  |  |
| Older adult has a car | Unmatched | 0.19 | 0.14 | 13.4 |  | 0.002 |
|  | Matched | 0.17 | 0.16 | 3.0 | 77.9 | 0.601 |
|  |  |  |  |  |  |  |
| Literacy | Unmatched | 0.35 | 0.30 | 10.2 |  | 0.018 |
|  | Matched | 0.31 | 0.31 | -0.7 | 93.4 | 0.904 |
|  |  |  |  |  |  |  |
| Total monthly household expenditure 1st quartile | Unmatched | 0.25 | 0.24 | 1.0 |  | 0.813 |
|  | Matched | 0.23 | 0.25 | -4.8 | -366.4 | 0.390 |
|  |  |  |  |  |  |  |
| Total monthly household expenditure 2nd quartile | Unmatched | 0.26 | 0.27 | -1.6 |  | 0.708 |
|  | Matched | 0.28 | 0.26 | 3.6 | -120.2 | 0.529 |
|  |  |  |  |  |  |  |
| Total monthly household expenditure 3rd quartile | Unmatched | 0.27 | 0.22 | 11.4 |  | 0.009 |
|  | Matched | 0.22 | 0.22 | 1.5 | 87.1 | 0.787 |
|  |  |  |  |  |  |  |
| Total monthly household expenditure 4th quartile | Unmatched | 0.22 | 0.27 | -10.7 |  | 0.013 |
|  | Matched | 0.27 | 0.27 | -0.4 | 96.6 | 0.950 |
|  |  |  |  |  |  |  |
| Locality deprivation index | Unmatched | -0.09 | -0.37 | 48.8 |  | <0.001 |
|  | Matched | -0.25 | -0.27 | 3.1 | 93.6 | 0.564 |

**Table S3. Results of matching using kernel algorithm**

|  | Matched | Treated | Control | Percentage of bias | Percentage of reduction on \|bias\| | p-value |
| --- | --- | --- | --- | --- | --- | --- |
| Female | Unmatched | 0.50 | 0.64 | -28.6 |  | <0.001 |
|  | Matched | 0.50 | 0.51 | -0.6 | 97.9 | 0.877 |
|  |  |  |  |  |  |  |
| Indigenous condition | Unmatched | 0.33 | 0.37 | -8.7 |  | 0.046 |
|  | Matched | 0.33 | 0.35 | -3.7 | 57.2 | 0.331 |
|  |  |  |  |  |  |  |
| Head of the household | Unmatched | 0.64 | 0.72 | -17.3 |  | <0.001 |
|  | Matched | 0.65 | 0.64 | 0.6 | 96.8 | 0.890 |
|  |  |  |  |  |  |  |
| Difficulties with basic activities of daily life | Unmatched | 0.18 | 0.20 | -5.8 |  | 0.175 |
|  | Matched | 0.18 | 0.17 | 1.9 | 67.9 | 0.617 |
|  |  |  |  |  |  |  |
| Difficulties with instrumental activities of daily life | Unmatched | 0.14 | 0.16 | -6.0 |  | 0.163 |
|  | Matched | 0.14 | 0.13 | 1.4 | 76.3 | 0.701 |
|  |  |  |  |  |  |  |
| Presence of a chronic condition | Unmatched | 0.50 | 0.53 | -4.8 |  | 0.265 |
|  | Matched | 0.50 | 0.49 | 2.3 | 53.0 | 0.559 |
|  |  |  |  |  |  |  |
| Paid job | Unmatched | 0.21 | 0.22 | -1.6 |  | 0.705 |
|  | Matched | 0.21 | 0.21 | 0.7 | 57.9 | 0.858 |
|  |  |  |  |  |  |  |
| Older adult living alone | Unmatched | 0.04 | 0.15 | -36.6 |  | <0.001 |
|  | Matched | 0.04 | 0.05 | -2.3 | 93.6 | 0.401 |
|  |  |  |  |  |  |  |
| Civil status | Unmatched | 0.65 | 0.46 | 38.7 |  | <0.001 |
|  | Matched | 0.65 | 0.64 | 2.7 | 93.2 | 0.485 |
|  |  |  |  |  |  |  |
| Unigeneracional household (just older adults living at home) | Unmatched | 0.21 | 0.35 | -30.4 |  | <0.001 |
|  | Matched | 0.21 | 0.21 | 0.1 | 99.7 | 0.982 |
|  |  |  |  |  |  |  |
| Older adult has someone who can be his/her economic support | Unmatched | 0.70 | 0.71 | -2.5 |  | 0.566 |
|  | Matched | 0.70 | 0.71 | -2.1 | 14.3 | 0.581 |
|  |  |  |  |  |  |  |
| Older adult has a house | Unmatched | 0.62 | 0.65 | -6.6 |  | 0.130 |
|  | Matched | 0.62 | 0.63 | -1.1 | 83.7 | 0.784 |
|  |  |  |  |  |  |  |
| Older adult has a car | Unmatched | 0.19 | 0.14 | 13.4 |  | 0.002 |
|  | Matched | 0.19 | 0.20 | -1.4 | 89.8 | 0.740 |
|  |  |  |  |  |  |  |
| Literacy | Unmatched | 0.35 | 0.30 | 10.2 |  | 0.018 |
|  | Matched | 0.35 | 0.32 | 5.9 | 42.2 | 0.130 |
|  |  |  |  |  |  |  |
| Total monthly household expenditure 1st quartile | Unmatched | 0.25 | 0.24 | 1.0 |  | 0.813 |
|  | Matched | 0.25 | 0.25 | -0.7 | 35.7 | 0.866 |
|  |  |  |  |  |  |  |
| Total monthly household expenditure 2nd quartile | Unmatched | 0.26 | 0.27 | -1.6 |  | 0.708 |
|  | Matched | 0.26 | 0.26 | 1.4 | 16.3 | 0.724 |
|  |  |  |  |  |  |  |
| Total monthly household expenditure 3rd quartile | Unmatched | 0.27 | 0.22 | 11.4 |  | 0.009 |
|  | Matched | 0.27 | 0.25 | 4.3 | 62.2 | 0.277 |
|  |  |  |  |  |  |  |
| Total monthly household expenditure 4th quartile | Unmatched | 0.22 | 0.27 | -10.7 |  | 0.013 |
|  | Matched | 0.22 | 0.24 | -5.0 | 53.0 | 0.188 |
|  |  |  |  |  |  |  |
| Locality deprivation index | Unmatched | -0.09 | -0.37 | 48.8 |  | <0.001 |
|  | Matched | -0.09 | -0.07 | -3.4 | 93.0 | 0.386 |

**Table S4. Testing the parallelism assumption: alternative control groups**

|  | **Original control group (OA 70-74, localities<2,500)** | **Alternative control group (OA 65-69, localities<2,500)** | **Alternative control group (OA 65-69, localities>2,500)** |
| --- | --- | --- | --- |
| Depressive symptoms (GDS≥6) | -0.050* [0.028] | -0.034 [0.024] | 0.012 [0.030] |
| Participates in making household decisions | 0.089*** [0.027] | -0.012 [0.024] | 0.040 [0.031] |
| Participates in household spending decisions | 0.106*** [0.029] | -0.008 [0.010] | 0.091** [0.030] |

GDS: Geriatric Depression Scale; OA: older adult

Linear probability models with fixed effect at individual level, adjusted for time-varying covariates in Table 1

Standard errors in brackets

*p < 0.10; **p < 0.05; *** p < 0.01

**Table S5. Testing the parallelism assumption: alternative outcomes**

|  | **Original control group (OA 70-74, localities<2,500)** |
| --- | --- |
| Older adults' number of children who are currently alive | 0.093 [0.080] |
| Number of years married or living with partner (for OA) | 0.339 [0.640] |
| Percentage of edentulous (for all members of the household) | -0.020 [0.016] |
| Percentage of hip fracture in the last 12 months (for OA) | -0.002 [0.007] |
| Death of ≥1 household member in the last 12 months | 0.005 [0.009] |
| Proportion of deaths in the household in the last 12 months | -0.003 [0.004] |
| Number of days ill or with health discomfort in the last 4 weeks before the interview (average for all members of the household, except the OA) | 0.545 [0.384] |
| Number of walking kilometers without tiring (average for all members of the household, except the OA) | -0.300 [1.119] |

OA: Older adult

Linear probability models with fixed effect at individual level, adjusted for time-varying covariates in Table 1

Standard errors in brackets

1. OA potential beneficiaries are all the adults who do not receive program benefits even though they are considered eligible in regards to the program requisites of age and residence in localities covered by the program *70 y más.* [↑](#footnote-ref-1)
2. Suspended OA beneficiaries is an emergent category and refers to those who were beneficiaries but were suspended from receiving payment for some administrative problem related to the program’s rules of operation. This category emerged grounded in fieldwork. [↑](#footnote-ref-2)
3. Key actors are those subjects who although not being a part of the main group of interest in the program have important knowledge of community successes and can offer an external perspective. [↑](#footnote-ref-3)
